# Addiction as a brain disease revised: why it still matters, and the need for consilience

**DOI:** 10.1038/s41386-020-00950-y

**Published:** 2021-02-22

**Authors:** Markus Heilig, James MacKillop, Diana Martinez, Jürgen Rehm, Lorenzo Leggio, Louk J. M. J. Vanderschuren

**Affiliations:** 1 Center for Social and Affective Neuroscience, Department of Biomedical and Clinical Sciences, Linköping University, Linköping, Sweden; 2 Peter Boris Centre for Addictions Research, McMaster University and St. Joseph’s Healthcare Hamilton, Hamilton, ON Canada; 3 Homewood Research Institute, Guelph, ON Canada; 4 New York State Psychiatric Institute and Columbia University Irving Medical Center, New York, NY USA; 5 Institute for Mental Health Policy Research & Campbell Family Mental Health Research Institute, Centre for Addiction and Mental Health (CAMH), Toronto, ON Canada; 6 Dalla Lana School of Public Health and Department of Psychiatry, University of Toronto (UofT), Toronto, ON Canada; 7 Klinische Psychologie & Psychotherapie, Technische Universität Dresden, Dresden, Germany; 8 Department of International Health Projects, Institute for Leadership and Health Management, I.M. Sechenov First Moscow State Medical University, Moscow, Russia; 9 Clinical Psychoneuroendocrinology and Neuropsychopharmacology Section, Translational Addiction Medicine Branch, National Institute on Drug Abuse Intramural Research Program and National Institute on Alcohol Abuse and Alcoholism Division of Intramural Clinical and Biological Research, National Institutes of Health, Baltimore and Bethesda, MD USA; 10 Department of Population Health Sciences, Unit Animals in Science and Society, Faculty of Veterinary Medicine, Utrecht University, Utrecht, the Netherlands

**Keywords:** Addiction, Addiction

## Abstract

The view that substance addiction is a brain disease, although widely accepted in the neuroscience community, has become subject to acerbic criticism in recent years. These criticisms state that the brain disease view is deterministic, fails to account for heterogeneity in remission and recovery, places too much emphasis on a compulsive dimension of addiction, and that a specific neural signature of addiction has not been identified. We acknowledge that some of these criticisms have merit, but assert that the foundational premise that addiction has a neurobiological basis is fundamentally sound. We also emphasize that denying that addiction is a brain disease is a harmful standpoint since it contributes to reducing access to healthcare and treatment, the consequences of which are catastrophic. Here, we therefore address these criticisms, and in doing so provide a contemporary update of the brain disease view of addiction. We provide arguments to support this view, discuss why apparently spontaneous remission does not negate it, and how seemingly compulsive behaviors can co-exist with the sensitivity to alternative reinforcement in addiction. Most importantly, we argue that the brain is the biological substrate from which both addiction and the capacity for behavior change arise, arguing for an intensified neuroscientific study of recovery. More broadly, we propose that these disagreements reveal the need for multidisciplinary research that integrates neuroscientific, behavioral, clinical, and sociocultural perspectives.

## Introduction

Close to a quarter of a century ago, then director of the US National Institute on Drug Abuse Alan Leshner famously asserted that “addiction is a brain disease”, articulated a set of implications of this position, and outlined an agenda for realizing its promise [1]. The paper, now cited almost 2000 times, put forward a position that has been highly influential in guiding the efforts of researchers, and resource allocation by funding agencies. A subsequent 2000 paper by McLellan et al. [2] examined whether data justify distinguishing addiction from other conditions for which a disease label is rarely questioned, such as diabetes, hypertension or asthma. It concluded that neither genetic risk, the role of personal choices, nor the influence of environmental factors differentiated addiction in a manner that would warrant viewing it differently; neither did relapse rates, nor compliance with treatment. The authors outlined an agenda closely related to that put forward by Leshner, but with a more clinical focus. Their conclusion was that addiction should be insured, treated, and evaluated like other diseases. This paper, too, has been exceptionally influential by academic standards, as witnessed by its ~3000 citations to date. What may be less appreciated among scientists is that its impact in the real world of addiction treatment has remained more limited, with large numbers of patients still not receiving evidence-based treatments.

In recent years, the conceptualization of addiction as a brain disease has come under increasing criticism. When first put forward, the brain disease view was mainly an attempt to articulate an effective response to prevailing nonscientific, moralizing, and stigmatizing attitudes to addiction. According to these attitudes, addiction was simply the result of a person’s moral failing or weakness of character, rather than a “real” disease [3]. These attitudes created barriers for people with substance use problems to access evidence-based treatments, both those available at the time, such as opioid agonist maintenance, cognitive behavioral therapy-based relapse prevention, community reinforcement or contingency management, and those that could result from research. To promote patient access to treatments, scientists needed to argue that there is a biological basis beneath the challenging behaviors of individuals suffering from addiction. This argument was particularly targeted to the public, policymakers and health care professionals, many of whom held that since addiction was a misery people brought upon themselves, it fell beyond the scope of medicine, and was neither amenable to treatment, nor warranted the use of taxpayer money.

Present-day criticism directed at the conceptualization of addiction as a brain disease is of a very different nature. It originates from within the scientific community itself, and asserts that this conceptualization is neither supported by data, nor helpful for people with substance use problems [4–8]. Addressing these critiques requires a very different perspective, and is the objective of our paper. We readily acknowledge that in some cases, recent critiques of the notion of addiction as a brain disease as postulated originally have merit, and that those critiques require the postulates to be re-assessed and refined. In other cases, we believe the arguments have less validity, but still provide an opportunity to update the position of addiction as a brain disease. Our overarching concern is that questionable arguments against the notion of addiction as a brain disease may harm patients, by impeding access to care, and slowing development of novel treatments.

A premise of our argument is that any useful conceptualization of addiction requires an understanding both of the brains involved, and of environmental factors that interact with those brains [9]. These environmental factors critically include availability of drugs, but also of healthy alternative rewards and opportunities. As we will show, stating that brain mechanisms are critical for understanding and treating addiction in no way negates the role of psychological, social and socioeconomic processes as both causes and consequences of substance use. To reflect this complex nature of addiction, we have assembled a team with expertise that spans from molecular neuroscience, through animal models of addiction, human brain imaging, clinical addiction medicine, to epidemiology. What brings us together is a passionate commitment to improving the lives of people with substance use problems through science and science-based treatments, with empirical evidence as the guiding principle.

**Fig. 1:**
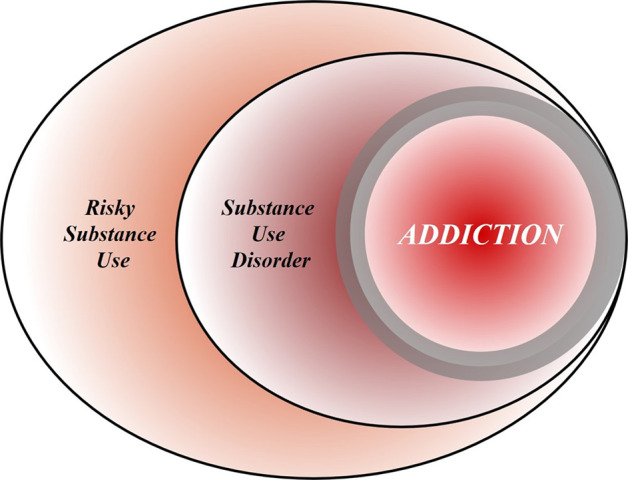
A heuristic Venn diagram of the putative relationships among risky (hazardous) substance use, substance use disorder (SUD), and addiction. Risky (hazardous) substance use refers to quantity/frequency indicators of consumption; SUD refers to individuals who meet criteria for a DSM-5 diagnosis (mild, moderate, or severe); and addiction refers to individuals who exhibit persistent difficulties with self-regulation of drug consumption. Among high-risk individuals, a subgroup will meet criteria for SUD and, among those who have an SUD, a further subgroup would be considered to be addicted to the drug. However, the boundary for addiction is intentionally blurred to reflect that the dividing line for defining addiction within the category of SUD remains an open empirical question.

To achieve this goal, we first discuss the nature of the disease concept itself, and why we believe it is important for the science and treatment of addiction. This is followed by a discussion of the main points raised when the notion of addiction as a brain disease has come under criticism. Key among those are claims that spontaneous remission rates are high; that a specific brain pathology is lacking; and that people suffering from addiction, rather than behaving “compulsively”, in fact show a preserved ability to make informed and advantageous choices. In the process of discussing these issues, we also address the common criticism that viewing addiction as a brain disease is a fully deterministic theory of addiction. For our argument, we use the term “addiction” as originally used by Leshner [1]; in Box 1, we map out and discuss how this construct may relate to the current diagnostic categories, such as Substance Use Disorder (SUD) and its different levels of severity (Fig. 1).

Box 1 What’s in a name? Differentiating hazardous use, substance use disorder, and addiction Although our principal focus is on the brain disease model of addiction, the definition of addiction itself is a source of ambiguity. Here, we provide a perspective on the major forms of terminology in the field.

Hazardous Substance Use

Hazardous (risky) substance use refers to quantitative levels of consumption that increase an individual’s risk for adverse health consequences. In practice, this pertains to alcohol use [110, 111]. Clinically, alcohol consumption that exceeds guidelines for moderate drinking has been used to prompt brief interventions or referral for specialist care [112]. More recently, a reduction in these quantitative levels has been validated as treatment endpoints [113].

Substance Use Disorder

SUD refers to the DSM-5 diagnosis category that encompasses significant impairment or distress resulting from specific categories of psychoactive drug use. The diagnosis of SUD is operationalized as 2 or more of 11 symptoms over the past year. As a result, the diagnosis is heterogenous, with more than 1100 symptom permutations possible. The diagnosis in DSM-5 is the result of combining two diagnoses from the DSM-IV, abuse and dependence, which proved to be less valid than a single dimensional approach [114]. Critically, SUD includes three levels of severity: mild (2–3 symptoms), moderate (4–5 symptoms), and severe (6+ symptoms). The International Classification of Diseases (ICD) system retains two diagnoses, harmful use (lower severity) and substance dependence (higher severity).

Addiction

Addiction is a natural language concept, etymologically meaning enslavement, with the contemporary meaning traceable to the Middle and Late Roman Republic periods [115]. As a scientific construct, drug addiction can be defined as a state in which an individual exhibits an inability to self-regulate consumption of a substance, although it does not have an operational definition. Regarding clinical diagnosis, as it is typically used in scientific and clinical parlance, addiction is not synonymous with the simple presence of SUD. Nowhere in DSM-5 is it articulated that the diagnostic threshold (or any specific number/type of symptoms) should be interpreted as reflecting addiction, which inherently connotes a high degree of severity. Indeed, concerns were raised about setting the diagnostic standard too low because of the issue of potentially conflating a low-severity SUD with addiction [116]. In scientific and clinical usage, addiction typically refers to individuals at a moderate or high severity of SUD. This is consistent with the fact that moderate-to-severe SUD has the closest correspondence with the more severe diagnosis in ICD [117–119]. Nonetheless, akin to the undefined overlap between hazardous use and SUD, the field has not identified the exact thresholds of SUD symptoms above which addiction would be definitively present.

Integration

The ambiguous relationships among these terms contribute to misunderstandings and disagreements. Figure 1 provides a simple working model of how these terms overlap. Fundamentally, we consider that these terms represent successive dimensions of severity, clinical “nesting dolls”. Not all individuals consuming substances at hazardous levels have an SUD, but a subgroup do. Not all individuals with a SUD are addicted to the drug in question, but a subgroup are. At the severe end of the spectrum, these domains converge (heavy consumption, numerous symptoms, the unambiguous presence of addiction), but at low severity, the overlap is more modest. The exact mapping of addiction onto SUD is an open empirical question, warranting systematic study among scientists, clinicians, and patients with lived experience. No less important will be future research situating our definition of SUD using more objective indicators (e.g., [55, 120]), brain-based and otherwise, and more precisely in relation to clinical needs [121]. Finally, such work should ultimately be codified in both the DSM and ICD systems to demarcate clearly where the attribution of addiction belongs within the clinical nosology, and to foster greater clarity and specificity in scientific discourse.

### What is a disease?

In his classic 1960 book “The Disease Concept of Alcoholism”, Jellinek noted that in the alcohol field, the debate over the disease concept was plagued by too many definitions of “alcoholism” and too few definitions of “disease” [10]. He suggested that the addiction field needed to follow the rest of medicine in moving away from viewing disease as an “entity”, i.e., something that has “its own independent existence, apart from other things” [11]. To modern medicine, he pointed out, a disease is simply a label that is agreed upon to describe a cluster of substantial, deteriorating changes in the structure or function of the human body, and the accompanying deterioration in biopsychosocial functioning. Thus, he concluded that alcoholism can simply be defined as changes in structure or function of the body due to drinking that cause disability or death. A disease label is useful to identify groups of people with commonly co-occurring constellations of problems—syndromes—that significantly impair function, and that lead to clinically significant distress, harm, or both. This convention allows a systematic study of the condition, and of whether group members benefit from a specific intervention.

It is not trivial to delineate the exact category of harmful substance use for which a label such as addiction is warranted (See Box 1). Challenges to diagnostic categorization are not unique to addiction, however. Throughout clinical medicine, diagnostic cut-offs are set by consensus, commonly based on an evolving understanding of thresholds above which people tend to benefit from available interventions. Because assessing benefits in large patient groups over time is difficult, diagnostic thresholds are always subject to debate and adjustments. It can be debated whether diagnostic thresholds “merely” capture the extreme of a single underlying population, or actually identify a subpopulation that is at some level distinct. Resolving this issue remains challenging in addiction, but once again, this is not different from other areas of medicine [see e.g., [12] for type 2 diabetes]. Longitudinal studies that track patient trajectories over time may have a better ability to identify subpopulations than cross-sectional assessments [13].

By this pragmatic, clinical understanding of the disease concept, it is difficult to argue that “addiction” is unjustified as a disease label. Among people who use drugs or alcohol, some progress to using with a quantity and frequency that results in impaired function and often death, making substance use a major cause of global disease burden [14]. In these people, use occurs with a pattern that in milder forms may be challenging to capture by current diagnostic criteria (See Box 1), but is readily recognized by patients, their families and treatment providers when it reaches a severity that is clinically significant [see [15] for a classical discussion]. In some cases, such as opioid addiction, those who receive the diagnosis stand to obtain some of the greatest benefits from medical treatments in all of clinical medicine [16, 17]. Although effect sizes of available treatments are more modest in nicotine [18] and alcohol addiction [19], the evidence supporting their efficacy is also indisputable. A view of addiction as a disease is justified, because it is beneficial: a failure to diagnose addiction drastically increases the risk of a failure to treat it [20].

Of course, establishing a diagnosis is not a requirement for interventions to be meaningful. People with hazardous or harmful substance use who have not (yet) developed addiction should also be identified, and interventions should be initiated to address their substance-related risks. This is particularly relevant for alcohol, where even in the absence of addiction, use is frequently associated with risks or harm to self, e.g., through cardiovascular disease, liver disease or cancer, and to others, e.g., through accidents or violence [21]. Interventions to reduce hazardous or harmful substance use in people who have not developed addiction are in fact particularly appealing. In these individuals, limited interventions are able to achieve robust and meaningful benefits [22], presumably because patterns of misuse have not yet become entrenched.

Thus, as originally pointed out by McLellan and colleagues, most of the criticisms of addiction as a disease could equally be applied to other medical conditions [2]. This type of criticism could also be applied to other psychiatric disorders, and that has indeed been the case historically [23, 24]. Today, there is broad consensus that those criticisms were misguided. Few, if any healthcare professionals continue to maintain that schizophrenia, rather than being a disease, is a normal response to societal conditions. Why, then, do people continue to question if addiction is a disease, but not whether schizophrenia, major depressive disorder or post-traumatic stress disorder are diseases? This is particularly troubling given the decades of data showing high co-morbidity of addiction with these conditions [25, 26]. We argue that it comes down to stigma. Dysregulated substance use continues to be perceived as a self-inflicted condition characterized by a lack of willpower, thus falling outside the scope of medicine and into that of morality [3].

### Chronic and relapsing, developmentally-limited, or spontaneously remitting?

Much of the critique targeted at the conceptualization of addiction as a brain disease focuses on its original assertion that addiction is a chronic and relapsing condition. Epidemiological data are cited in support of the notion that large proportions of individuals achieve remission [27], frequently without any formal treatment [28, 29] and in some cases resuming low risk substance use [30]. For instance, based on data from the National Epidemiologic Survey on Alcohol and Related Conditions (NESARC) study [27], it has been pointed out that a significant proportion of people with an addictive disorder quit each year, and that most afflicted individuals ultimately remit. These spontaneous remission rates are argued to invalidate the concept of a chronic, relapsing disease [4].

Interpreting these and similar data is complicated by several methodological and conceptual issues. First, people may appear to remit spontaneously because they actually do, but also because of limited test–retest reliability of the diagnosis [31]. For instance, using a validated diagnostic interview and trained interviewers, the Collaborative Studies on Genetics of Alcoholism examined the likelihood that an individual diagnosed with a *lifetime history* of substance dependence would retain this classification after 5 years. This is obviously a diagnosis that, once met, by definition cannot truly remit. Lifetime alcohol dependence was indeed stable in individuals recruited from addiction treatment units, ~90% for women, and 95% for men. In contrast, in a community-based sample similar to that used in the NESARC [27], stability was only ~30% and 65% for women and men, respectively. The most important characteristic that determined diagnostic stability was severity. Diagnosis was stable in severe, treatment-seeking cases, but not in general population cases of alcohol dependence.

These data suggest that commonly used diagnostic criteria alone are simply over-inclusive for a reliable, clinically meaningful diagnosis of addiction. They do identify a core group of treatment seeking individuals with a reliable diagnosis, but, if applied to nonclinical populations, also flag as “cases” a considerable halo of individuals for whom the diagnostic categorization is unreliable. Any meaningful discussion of remission rates needs to take this into account, and specify which of these two populations that is being discussed. Unfortunately, the DSM-5 has not made this task easier. With only 2 out of 11 symptoms being sufficient for a diagnosis of SUD, it captures under a single diagnostic label individuals in a “mild” category, whose diagnosis is likely to have very low test–retest reliability, and who are unlikely to exhibit a chronic relapsing course, together with people at the severe end of the spectrum, whose diagnosis is reliable, many of whom do show a chronic relapsing course.

The NESARC data nevertheless show that close to 10% of people in the general population who are diagnosed with alcohol addiction (here equated with DSM-IV “dependence” used in the NESARC study) *never* remitted throughout their participation in the survey. The base life-time prevalence of alcohol dependence in NESARC was 12.5% [32]. Thus, the data cited against the concept of addiction as a chronic relapsing disease in fact indicate that over 1% of the US population develops an alcohol-related condition that is associated with high morbidity and mortality, and whose chronic and/or relapsing nature cannot be disputed, since it does not remit.

Secondly, the analysis of NESARC data [4, 27] omits opioid addiction, which, together with alcohol and tobacco, is the largest addiction-related public health problem in the US [33]. This is probably the addictive condition where an analysis of cumulative evidence most strikingly supports the notion of a chronic disorder with frequent relapses in a large proportion of people affected [34]. Of course, a large number of people with opioid addiction are unable to express the chronic, relapsing course of their disease, because over the long term, their mortality rate is about 15 times greater than that of the general population [35]. However, even among those who remain alive, the prevalence of stable abstinence from opioid use after 10–30 years of observation is <30%. Remission may not always require abstinence, for instance in the case of alcohol addiction, but is a reasonable proxy for remission with opioids, where return to controlled use is rare. Embedded in these data is a message of literally vital importance: when opioid addiction is diagnosed and treated as a chronic relapsing disease, outcomes are markedly improved, and retention in treatment is associated with a greater likelihood of abstinence.

The fact that significant numbers of individuals exhibit a chronic relapsing course does not negate that even larger numbers of individuals with SUD according to current diagnostic criteria do not. For instance, in many countries, the highest prevalence of substance use problems is found among young adults, aged 18–25 [36], and a majority of these ‘age out’ of excessive substance use [37]. It is also well documented that many individuals with SUD achieve longstanding remission, in many cases without any formal treatment (see e.g., [27, 30, 38]).

Collectively, the data show that the course of SUD, as defined by current diagnostic criteria, is highly heterogeneous. Accordingly, we do not maintain that a chronic relapsing course is a defining feature of SUD. When present in a patient, however, such as course is of clinical significance, because it identifies a need for long-term disease management [2], rather than expectations of a recovery that may not be within the individual’s reach [39]. From a conceptual standpoint, however, a chronic relapsing course is neither necessary nor implied in a view that addiction is a brain disease. This view also does not mean that it is irreversible and hopeless. Human neuroscience documents restoration of functioning after abstinence [40, 41] and reveals predictors of clinical success [42]. If anything, this evidence suggests a need to increase efforts devoted to neuroscientific research on addiction recovery [40, 43].

### Lessons from genetics

For alcohol addiction, meta-analysis of twin and adoption studies has estimated heritability at ~50%, while estimates for opioid addiction are even higher [44, 45]. Genetic risk factors are to a large extent shared across substances [46]. It has been argued that a genetic contribution cannot support a disease view of a behavior, because most behavioral traits, including religious and political inclinations, have a genetic contribution [4]. This statement, while correct in pointing out broad heritability of behavioral traits, misses a fundamental point. Genetic architecture is much like organ structure. The fact that normal anatomy shapes healthy organ function does not negate that an altered structure can contribute to pathophysiology of disease. The structure of the genetic landscape is no different. Critics further state that a “genetic predisposition is not a recipe for compulsion”, but no neuroscientist or geneticist would claim that genetic risk is “a recipe for compulsion”. Genetic risk is probabilistic, not deterministic. However, as we will see below, in the case of addiction, it contributes to large, consistent probability shifts towards maladaptive behavior.

In dismissing the relevance of genetic risk for addiction, Hall writes that “a large number of alleles are involved in the genetic susceptibility to addiction and individually these alleles might very weakly predict a risk of addiction”. He goes on to conclude that “generally, genetic prediction of the risk of disease (even with whole-genome sequencing data) is unlikely to be informative for most people who have a so-called average risk of developing an addiction disorder” [7]. This reflects a fundamental misunderstanding of polygenic risk. It is true that a large number of risk alleles are involved, and that the explanatory power of currently available polygenic risk scores for addictive disorders lags behind those for e.g., schizophrenia or major depression [47, 48]. The only implication of this, however, is that low average effect sizes of risk alleles in addiction necessitate larger study samples to construct polygenic scores that account for a large proportion of the known heritability.

However, a heritability of addiction of ~50% indicates that DNA sequence variation accounts for 50% of the risk for this condition. Once whole genome sequencing is readily available, it is likely that it will be possible to identify most of that DNA variation. For clinical purposes, those polygenic scores will of course not replace an understanding of the intricate web of biological and social factors that promote or prevent expression of addiction in an individual case; rather, they will add to it [49]. Meanwhile, however, genome-wide association studies in addiction have already provided important information. For instance, they have established that the genetic underpinnings of alcohol addiction only partially overlap with those for alcohol consumption, underscoring the genetic distinction between pathological and nonpathological drinking behaviors [50].

It thus seems that, rather than negating a rationale for a disease view of addiction, the important implication of the polygenic nature of addiction risk is a very different one. Genome-wide association studies of complex traits have largely confirmed the century old “infinitisemal model” in which Fisher reconciled Mendelian and polygenic traits [51]. A key implication of this model is that genetic susceptibility for a complex, polygenic trait is continuously distributed in the population. This may seem antithetical to a view of addiction as a distinct disease category, but the contradiction is only apparent, and one that has long been familiar to quantitative genetics. Viewing addiction *susceptibility* as a polygenic quantitative trait, and addiction as a disease category is entirely in line with Falconer’s theorem, according to which, in a given set of environmental conditions, a certain level of genetic susceptibility will determine a threshold above which disease will arise.

### A brain disease? Then show me the brain lesion!

The notion of addiction as a brain disease is commonly criticized with the argument that a specific pathognomonic brain lesion has not been identified. Indeed, brain imaging findings in addiction (perhaps with the exception of extensive neurotoxic gray matter loss in advanced alcohol addiction) are nowhere near the level of specificity and sensitivity required of clinical diagnostic tests. However, this criticism neglects the fact that neuroimaging is not used to diagnose many neurologic and psychiatric disorders, including epilepsy, ALS, migraine, Huntington’s disease, bipolar disorder, or schizophrenia. Even among conditions where signs of disease can be detected using brain imaging, such as Alzheimer’s and Parkinson’s disease, a scan is best used in conjunction with clinical acumen when making the diagnosis. Thus, the requirement that addiction be detectable with a brain scan in order to be classified as a disease does not recognize the role of neuroimaging in the clinic.

For the foreseeable future, the main objective of imaging in addiction research is not to diagnose addiction, but rather to improve our understanding of mechanisms that underlie it. The hope is that mechanistic insights will help bring forward new treatments, by identifying candidate targets for them, by pointing to treatment-responsive biomarkers, or both [52]. Developing innovative treatments is essential to address unmet treatment needs, in particular in stimulant and cannabis addiction, where no approved medications are currently available. Although the task to develop novel treatments is challenging, promising candidates await evaluation [53]. A particular opportunity for imaging-based research is related to the complex and heterogeneous nature of addictive disorders. Imaging-based biomarkers hold the promise of allowing this complexity to be deconstructed into specific functional domains, as proposed by the RDoC initiative [54] and its application to addiction [55, 56]. This can ultimately guide the development of personalized medicine strategies to addiction treatment.

Countless imaging studies have reported differences in brain structure and function between people with addictive disorders and those without them. Meta-analyses of structural data show that alcohol addiction is associated with gray matter losses in the prefrontal cortex, dorsal striatum, insula, and posterior cingulate cortex [57], and similar results have been obtained in stimulant-addicted individuals [58]. Meta-analysis of functional imaging studies has demonstrated common alterations in dorsal striatal, and frontal circuits engaged in reward and salience processing, habit formation, and executive control, across different substances and task-paradigms [59]. Molecular imaging studies have shown that large and fast increases in dopamine are associated with the reinforcing effects of drugs of abuse, but that after chronic drug use and during withdrawal, brain dopamine function is markedly decreased and that these decreases are associated with dysfunction of prefrontal regions [60]. Collectively, these findings have given rise to a widely held view of addiction as a disorder of fronto-striatal circuitry that mediates top-down regulation of behavior [61].

Critics reply that none of the brain imaging findings are sufficiently specific to distinguish between addiction and its absence, and that they are typically obtained in cross-sectional studies that can at best establish correlative rather than causal links. In this, they are largely right, and an updated version of a conceptualization of addiction as a brain disease needs to acknowledge this. Many of the structural brain findings reported are not specific for addiction, but rather shared across psychiatric disorders [62]. Also, for now, the most sophisticated tools of human brain imaging remain crude in face of complex neural circuit function. Importantly however, a vast literature from animal studies also documents functional changes in fronto-striatal circuits, as well their limbic and midbrain inputs, associated with addictive behaviors [63–68]. These are circuits akin to those identified by neuroimaging studies in humans, implicated in positive and negative emotions, learning processes and executive functions, altered function of which is thought to underlie addiction. These animal studies, by virtue of their cellular and molecular level resolution, and their ability to establish causality under experimental control, are therefore an important complement to human neuroimaging work.

Nevertheless, factors that seem remote from the activity of brain circuits, such as policies, substance availability and cost, as well as socioeconomic factors, also are critically important determinants of substance use. In this complex landscape, is the brain really a defensible focal point for research and treatment? The answer is “yes”. As powerfully articulated by Francis Crick [69], “You, your joys and your sorrows, your memories and your ambitions, your sense of personal identity and free will, are in fact no more than the behavior of a vast assembly of nerve cells and their associated molecules”. Social and interpersonal factors are critically important in addiction, but they can only exert their influences by impacting neural processes. They must be encoded as sensory data, represented together with memories of the past and predictions about the future, and combined with representations of interoceptive and other influences to provide inputs to the valuation machinery of the brain. Collectively, these inputs drive action selection and execution of behavior—say, to drink or not to drink, and then, within an episode, to stop drinking or keep drinking. Stating that the pathophysiology of addiction is largely about the brain does not ignore the role of other influences. It is just the opposite: it is attempting to understand *how* those important influences contribute to drug seeking and taking in the context of the brain, and vice versa.

But if the criticism is one of emphasis rather than of principle—i.e., too much brain, too little social and environmental factors – then neuroscientists need to acknowledge that they are in part guilty as charged. Brain-centric accounts of addiction have for a long time failed to pay enough attention to the inputs that social factors provide to neural processing behind drug seeking and taking [9]. This landscape is, however, rapidly changing. For instance, using animal models, scientists are finding that lack of social play early in life increases the motivation to take addictive substances in adulthood [70]. Others find that the opportunity to interact with a fellow rat is protective against addiction-like behaviors [71]. In humans, a relationship has been found between perceived social support, socioeconomic status, and the availability of dopamine D2 receptors [72, 73], a biological marker of addiction vulnerability. Those findings in turn provided translation of data from nonhuman primates, which showed that D2 receptor availability can be altered by changes in social hierarchy, and that these changes are associated with the motivation to obtain cocaine [74].

Epidemiologically, it is well established that social determinants of health, including major racial and ethnic disparities, play a significant role in the risk for addiction [75, 76]. Contemporary neuroscience is illuminating how those factors penetrate the brain [77] and, in some cases, reveals pathways of resilience [78] and how evidence-based prevention can interrupt those adverse consequences [79, 80]. In other words, from our perspective, viewing addiction as a brain disease in no way negates the importance of social determinants of health or societal inequalities as critical influences. In fact, as shown by the studies correlating dopamine receptors with social experience, imaging is capable of capturing the impact of the social environment on brain function. This provides a platform for understanding how those influences become embedded in the biology of the brain, which provides a biological roadmap for prevention and intervention.

We therefore argue that a contemporary view of addiction as a brain disease does not deny the influence of social, environmental, developmental, or socioeconomic processes, but rather proposes that the brain is the underlying material substrate upon which those factors impinge and from which the responses originate. Because of this, neurobiology is a critical level of analysis for understanding addiction, although certainly not the only one. It is recognized throughout modern medicine that a host of biological and non-biological factors give rise to disease; understanding the biological pathophysiology is critical for understanding etiology and informing treatment.

### Is a view of addiction as a brain disease deterministic?

A common criticism of the notion that addiction is a brain disease is that it is reductionist and in the end therefore deterministic [81, 82]. This is a fundamental misrepresentation. As indicated above, viewing addiction as a brain disease simply states that neurobiology is an undeniable component of addiction. A reason for deterministic interpretations may be that modern neuroscience emphasizes an understanding of proximal causality within research designs (e.g., whether an observed link between biological processes is mediated by a specific mechanism). That does not in any way reflect a superordinate assumption that neuroscience will achieve global causality. On the contrary, since we realize that addiction involves interactions between biology, environment and society, ultimate (complete) prediction of behavior based on an understanding of neural processes alone is neither expected, nor a goal.

A fairer representation of a contemporary neuroscience view is that it believes insights from neurobiology allow useful probabilistic models to be developed of the inherently stochastic processes involved in behavior [see [83] for an elegant recent example]. Changes in brain function and structure in addiction exert a powerful probabilistic influence over a person’s behavior, but one that is highly multifactorial, variable, and thus stochastic. Philosophically, this is best understood as being aligned with indeterminism, a perspective that has a deep history in philosophy and psychology [84]. In modern neuroscience, it refers to the position that the dynamic complexity of the brain, given the probabilistic threshold-gated nature of its biology (e.g., action potential depolarization, ion channel gating), means that behavior cannot be definitively predicted in any individual instance [85, 86].

### Driven by compulsion, or free to choose?

A major criticism of the brain disease view of addiction, and one that is related to the issue of determinism vs indeterminism, centers around the term “compulsivity” [6, 87–90] and the different meanings it is given. Prominent addiction theories state that addiction is characterized by a transition from controlled to “compulsive” drug seeking and taking [91–95], but allocate somewhat different meanings to “compulsivity”. By some accounts, compulsive substance use is habitual and insensitive to its outcomes [92, 94, 96]. Others refer to compulsive use as a result of increasing incentive value of drug associated cues [97], while others view it as driven by a recruitment of systems that encode negative affective states [95, 98].

The prototype for compulsive behavior is provided by obsessive-compulsive disorder (OCD), where compulsion refers to repeatedly and stereotypically carrying out actions that in themselves may be meaningful, but lose their purpose and become harmful when performed in excess, such as persistent handwashing until skin injuries result. Crucially, this happens despite a conscious desire to do otherwise. Attempts to resist these compulsions result in increasing and ultimately intractable anxiety [99]. This is in important ways different from the meaning of compulsivity as commonly used in addiction theories. In the addiction field, compulsive drug use typically refers to inflexible, drug-centered behavior in which substance use is insensitive to adverse consequences [100]. Although this phenomenon is not necessarily present in every patient, it reflects important symptoms of clinical addiction, and is captured by several DSM-5 criteria for SUD [101]. Examples are needle-sharing despite knowledge of a risk to contract HIV or Hepatitis C, drinking despite a knowledge of having liver cirrhosis, but also the neglect of social and professional activities that previously were more important than substance use. While these behaviors do show similarities with the compulsions of OCD, there are also important differences. For example, “compulsive” substance use is not necessarily accompanied by a conscious desire to withhold the behavior, nor is addictive behavior consistently impervious to change.

Critics question the existence of compulsivity in addiction altogether [5–7, 89], typically using a literal interpretation, i.e., that a person who uses alcohol or drugs simply can not do otherwise. Were that the intended meaning in theories of addiction—which it is not—it would clearly be invalidated by observations of preserved sensitivity of behavior to contingencies in addiction. Indeed, substance use is influenced both by the availability of alternative reinforcers, and the state of the organism. The roots of this insight date back to 1940, when Spragg found that chimpanzees would normally choose a banana over morphine. However, when physically dependent and in a state of withdrawal, their choice preference would reverse [102]. The critical role of alternative reinforcers was elegantly brought into modern neuroscience by Ahmed et al., who showed that rats extensively trained to self-administer cocaine would readily forego the drug if offered a sweet solution as an alternative [103]. This was later also found to be the case for heroin [103], methamphetamine [104] and alcohol [105]. Early residential laboratory studies on alcohol use disorder indeed revealed orderly operant control over alcohol consumption [106]. Furthermore, efficacy of treatment approaches such as contingency management, which provides systematic incentives for abstinence [107], supports the notion that behavioral choices in patients with addictions remain sensitive to reward contingencies.

Evidence that a capacity for choosing advantageously is preserved in addiction provides a valid argument against a narrow concept of “compulsivity” as rigid, immutable behavior that applies to all patients. It does not, however, provide an argument against addiction as a brain disease. If not from the brain, from where do the healthy and unhealthy choices people make originate? The critical question is whether addictive behaviors—for the most part—result from healthy brains responding normally to externally determined contingencies; or rather from a pathology of brain circuits that, through probabilistic shifts, promotes the likelihood of maladaptive choices even when reward contingencies are within a normal range. To resolve this question, it is critical to understand that the ability to choose advantageously is not an all-or-nothing phenomenon, but rather is about probabilities and their shifts, multiple faculties within human cognition, and their interaction. Yes, it is clear that most people whom we would consider to suffer from addiction remain able to choose advantageously much, if not most, of the time. However, it is also clear that the probability of them choosing to their own disadvantage, even when more salutary options are available and sometimes at the expense of losing their life, is systematically and quantifiably increased. There is a freedom of choice, yet there is a shift of prevailing choices that nevertheless can kill.

Synthesized, the notion of addiction as a disease of choice and addiction as a brain disease can be understood as two sides of the same coin. Both of these perspectives are informative, and they are complementary. Viewed this way, addiction is a brain disease in which a person’s choice faculties become profoundly compromised. To articulate it more specifically, embedded in and principally executed by the central nervous system, addiction can be understood as a disorder of choice preferences, preferences that overvalue immediate reinforcement (both positive and negative), preferences for drug-reinforcement in spite of costs, and preferences that are unstable (*“I’ll never drink like that again;” “this will be my last cigarette”*), prone to reversals in the form of lapses and relapse. From a contemporary neuroscience perspective, pre-existing vulnerabilities and persistent drug use lead to a vicious circle of substantive disruptions in the brain that impair and undermine choice capacities for adaptive behavior, but do not annihilate them. Evidence of generally intact decision making does not fundamentally contradict addiction as a brain disease.

## Conclusions

The present paper is a response to the increasing number of criticisms of the view that addiction is a chronic relapsing brain disease. In many cases, we show that those criticisms target tenets that are neither needed nor held by a contemporary version of this view. Common themes are that viewing addiction as a brain disease is criticized for being both too narrow (addiction is *only* a brain disease; no other perspectives or factors are important) or too far reaching (it purports to discover the *final* causes of addiction). With regard to disease course, we propose that viewing addiction as a chronic relapsing disease is appropriate for some populations, and much less so for others, simply necessitating better ways of delineating the populations being discussed. We argue that when considering addiction as a disease, the lens of neurobiology is valuable to use. It is not the only lens, and it does not have supremacy over other scientific approaches. We agree that critiques of neuroscience are warranted [108] and that critical thinking is essential to avoid deterministic language and scientific overreach.

Beyond making the case for a view of addiction as a brain disease, perhaps the more important question is when a specific level of analysis is most useful. For understanding the biology of addiction and designing biological interventions, a neurobiological view is almost certainly the most appropriate level of analysis, in particular when informed by an understanding of the behavioral manifestations. In contrast, for understanding the psychology of addiction and designing psychological interventions, behavioral science is the natural realm, but one that can often benefit from an understanding of the underlying neurobiology. For designing policies, such as taxation and regulation of access, economics and public administration provide the most pertinent perspectives, but these also benefit from biological and behavioral science insights.

Finally, we argue that progress would come from integration of these scientific perspectives and traditions. E.O. Wilson has argued more broadly for greater consilience [109], unity of knowledge, in science. We believe that addiction is among the areas where consilience is most needed. A plurality of disciplines brings important and trenchant insights to bear on this condition; it is the exclusive remit of no single perspective or field. Addiction inherently and necessarily requires multidisciplinary examination. Moreover, those who suffer from addiction will benefit most from the application of the full armamentarium of scientific perspectives.

## Funding and disclosures

Supported by the Swedish Research Council grants 2013-07434, 2019-01138 (MH); Netherlands Organisation for Health Research and Development (ZonMw) under project number 912.14.093 (LJMJV); NIDA and NIAAA intramural research programs (LL; the content is solely the responsibility of the author and does not necessarily represent the official views of the National Institutes of Health); the Peter Boris Chair in Addictions Research, Homewood Research Institute, and the National Institute on Alcohol Abuse and Alcoholism grants AA025911, AA024930, AA025849, AA027679 (JM; the content is solely the responsibility of the author and does not necessarily represent the official views of the National Institutes of Health).

MH has received consulting fees, research support or other compensation from Indivior, Camurus, BrainsWay, Aelis Farma, and Janssen Pharmaceuticals. JM is a Principal and Senior Scientist at BEAM Diagnostics, Inc. DM, JR, LL, and LJMJV declare no conflict of interest.
